# The effect of water molecules on paraquat salts: from physicochemical properties to environmental impact in the Brazilian Cerrado

**DOI:** 10.3389/fchem.2023.1267634

**Published:** 2023-09-19

**Authors:** Antônio S. N. Aguiar, Luiz B. Costa, Igor D. Borges, Gerardo Aguirre, Francisco L. Tejerina-Garro, Sandro Dutra e Silva, Hamilton B. Napolitano

**Affiliations:** ^1^ Programa de Pós-Graduação em Recursos Naturais do Cerrado, Universidade Estadual de Goiás, Anápolis, Brazil; ^2^ Programa de Pós-Graduação em Sociedade, Tecnologia e Meio Ambiente, Universidade Evangélica de Goiás, Anápolis, Brazil; ^3^ Centro de Graduados e Investigación en Química, Tecnológico Nacional de Mexico, Tijuana, Mexico; ^4^ Escola de Ciências Médicas e da Vida, Pontifícia Universidade Católica de Goiás, Goiânia, Brazil

**Keywords:** Paraquat, physicochemical properties, green revolution, Brazilian Cerrado, herbicide

## Abstract

**Introduction:** The green revolution model that is followed in the Brazilian Cerrado is dependent on mechanization, chemical fertilization for soil dressing and correction, and the use of herbicides. Paraquat is a methyl viologen herbicide marketed as bipyridylium dichloride salts and used (in low doses) to combat weeds in their post-emergence stage. It is a non-selective pesticide that causes the peroxidation of the lipids that make up the cell membrane, and when it comes into contact with foliage, it results in the death of the plant.

**Methods:** The effect of water molecules co-crystallized in Paraquat salt structures was analyzed in anhydrous, dihydrate, and trihydrate forms to understand those physicochemical properties in its redox activity. The frontier molecular orbitals were also carried out using DFT to obtain the chemical reactivity of the bipyridylium cation. Finally, the supramolecular arrangements were evaluated to analyze the physicochemical stability and acquire insights on superoxide anions.

**Results and discussion:** The electronic structure indicated that the BP cation presents an acidic character due to its low ELUMO value, while the salt has a more basic character due to its high EHOMO value. For this reason, the BP ion is more susceptible to reduction during the weeds’ photosynthesis process. During the process of plant photosynthesis, PQ is reduced to form a stable radical cation. In the supramolecular arrangement, the presence of water molecules increases the number of strong H-bonds, while the weak/moderate H-bonds are stabilized. PQ’s toxic effects are observed in wildlife, domesticated animals, human populations, and ecosystems. The influence of PQ on the terrestrial environment is limited because of the soil adsorption capacity associated with good agricultural practices. The current use of good agricultural practices in the Cerrado seems not to prevent the environmental impacts of herbicides like PQ because it aims for the expansion and profitability of large-scale farming based on input-intensive practices instead of sustainable agriculture processes.

## 1 Introduction

Paraquat (PQ) is a herbicide available in the *1,1'-dimethyl-4,4'-bipyridilium chloride* salt form. It is a methyl viologen compound first described in 1882, with redox properties discovered only in 1933 (Michaelis et al., 1933), and herbicidal properties described in 1958 (Brian et al., 1958). From then on, PQ began to be developed for commercial purposes, becoming available on the agricultural market in 1962. It is a non-selective, fast-acting contact herbicide used to control a broad spectrum of broadleaf weeds and grasses in sugarcane (Aekrathok et al., 2021), soybean (da Silva et al., 2021), cotton (Ferreira et al., 2018), rice (Lima et al., 2018), coffee (de Queiroz et al., 2018) and in fruit such as grapes, apples, and pineapples. PQ is listed under a pesticide category in regulatory classifications (*e.g.*, United States Environmental Protection Agency) due to its primary use.

As bipyridylium (BP) salt, PQ interrupts photosynthesis processes in plants, so that the main effect observed is the burning of plant tissue after exposure to light. This is because its mechanism of action consists of the electronic competition of the herbicide with photosystem I (PSI) ferredoxin present in chloroplasts during plant photosynthesis (Fukushima et al., 2002). PQ is reduced by NADPH-cytochrome *c* reductase, producing viologen methyl radicals that are instantly oxidized with O_2_—forming the superoxide radical (O_2_ ∙ ^–^), by cytochrome P-450 in the presence of tertiary amine N-oxides. In addition, other toxic oxygen species, including the hydroxyl radical (OH ∙ ), hydrogen peroxide (HOO ∙ ), and singlet oxygen (^1^O_2_), are formed, causing peroxidation of the lipids that constitute the cytoplasmic membrane, resulting in water loss and rapid desiccation of the plant, leading to its death (Dodge, 1982; Fukushima et al., 2002; Cui et al., 2019).

PQ is applied during the post-emergence stage of weeds (Cui et al., 2019). It is rapidly absorbed by the soil, undergoing a sorption process primarily driven by ion exchange, leading to deactivation (Weber and Weed, 1968). This herbicide can enter the aquatic environment via vertical transport through the soil profile (dissolved organic matter colloids and dispersal colloidal clay) (Santos et al., 2013) or runoff during the rainfall season (Veríssimo et al., 2018). This herbicide is highly soluble in water (561–700 g/L) (Tsai, 2013; Huang et al., 2019), but in waterbodies, it tends to be adsorbed by particles and sediment, displaying a half-life time between 2 and 820 years, depending on sunlight and water depth (Thi Hue et al., 2018). PQ has been found in surface and underground water, the former involving a potential source for drinking water contamination (Rial-Otero et al., 2006; Santos et al., 2013). In aqueous solutions, PQ can be photochemically degraded in the presence of oxygen and ultraviolet radiation (Tsai, 2013).

The Cerrado–*a neotropical savanna*–is the second largest Brazilian biome, encompassing originally about two million km^2^ (Oliveira and Marquis, 2002). This biome has been used since the Brazilian green revolution, forming the main agricultural Frontier and becoming one of the global centers for the production of grains and commodities (Michaelis et al., 1933; Brian et al., 1958; Dutra e Silva, 2023). In Brazil, the technique of choice since 1980 had been no-till agriculture, accompanied by the use of herbicides, mainly PQ, until 2020, when its use was banned (Ofstehage and Nehring, 2021). The green revolution model followed in Brazil has been based on a pattern of mechanization, chemical fertilization for soil dressing and correction, in addition to the use of *pesticides* to control pests and insects. In recent years, the country has stood out as one of the main import markets for pesticides, many of which are banned in their own countries of origin, especially by the European Union (Cabette et al., 2020; Rocha et al., 2022a; Rocha et al., 2022b). The discussion on *control* and/or *flexibility* in the use of pesticides in Brazil is associated with the context of the green revolution in the country (Glaeser, 2010; Paumgartten, 2020).

PQ is an example of the controversies and struggles among those who are in favor of or against the greater release of pesticides in Brazilian agriculture (Brazil, 2020). This issue is still complex, and there is no consensus on the *risks* and *benefits* of using PQ in agricultural production (Brown et al., 2004; Shoham, 2013). Few studies have been conducted about the impacts of PQ on the Cerrado biome: Lajmanovich (Lajmanovich et al., 1998) concludes that the tadpole *Scinax nasica* present in Cerrado regions underwent increased mortality when exposed to 30.0 and 50.0 mg PQ/L. Peruzzolo (Peruzzolo et al., 2021) indicate that the ingestion of PQ increases the mortality of *Scaptotrigona bipunctata*, a native bee found in the Cerrado; Lundberg (Lundberg, 2021) considered the use of herbicides in soybean crops between 2016 and 2018 and points out that PQ displays a very high potential impact on freshwater species because of the high value of its ecotoxicity, as measured by chemical toxic unit (CTU per kg released). Finally, the Brazilian ban on PQ use was based on its mutagenic potential in human germ cells in contact with this herbicide.

In this work, the effects of water molecules on the crystalline structures of PQ salts were described. Theoretical calculations were carried out using density functional theory (DFT) (Hohenberg and Kohn, 1964; Kohn and Sham, 1965), where the cation molecular and electronic structures of BP were analyzed. The chemical reactivity descriptors were obtained from Frontier molecular orbitals (FMO) (Zhang and Musgrave, 2007) to understand the influence of Cl^−^anions in the vicinity of the cation and simulate the effects on the cell environment. Furthermore, the physicochemical information on the capture of electrons during the herbicide’s action in the photosynthetic processes of plants (Fukushima et al., 2002) was obtained based on the spin density (Overhauser, 1962; Jacob and Reiher, 2012). Finally, the supramolecular arrangements of the anhydrous, dihydrate, and trihydrate PQ salts were analyzed on a physicochemical basis and associated with environmental impact in the Brazilian Cerrado.

## 2 Methods

### 2.1 Molecular modeling

The crystal structures of the PQ salts (1,1'-dimethyl-4,4'-bipyridylium dichloride), in anhydrous (PQC-I) (Russell and Wallwork, 1972), dihydrate (PQC-II) (Cousson et al., 1993), and trihydrate (PQC-III) (Argay and Kálmán, 1995) forms were obtained from the Cambridge Crystallographic Data Centre (CCDC) (Cambridge Crystallographic Data Centre, 2023), under codes 1228234, 1170961, and 110220, respectively. The crystal structure data of the salts is presented in Table 1, and the structural patterns were analyzed in the Mercury program (Macrae et al., 2006; Macrae et al., 2008). Theoretical calculations were carried out by DFT (Hohenberg and Kohn, 1964; Kohn and Sham, 1965), implemented in the Gaussian 16 program package (Frisch et al., 2016). For the calculations, the hybrid exchange-correlation functional with long-range correction, M06-2X (Zhao and Truhlar, 2008), combined with the basis set 6-311++G(d,p), in gas phase, was used. By the FMO energies (Zhang and Musgrave, 2007), the highest occupied molecular orbital (HOMO) and lowest unoccupied molecular orbital (LUMO), it was possible to compare the electronic structures of the BP cation and its respective salt, as well as to infer information about their chemical reactivity and kinetic stability. Spin density calculations (Overhauser, 1962; Jacob and Reiher, 2012) were also carried out to obtain information about the radical formed during the mechanism of action of the herbicide (Fukushima et al., 2002) on the photosystems of the weed.

**TABLE 1:** Crystallographic data and structure refinement for PQC-I, PQC-II, and PQC-III.

Crystal data	PQC-I	PQC-II	PQC-III
Chemical formula	C_12_H_14_N_2_Cl_2_	C_12_H_14_N_2_Cl_2_ ∙ 2H_2_O	C_12_H_14_N_2_Cl_2_ ∙ 3H_2_O
Molecular weight (g/mol)	257.158	293.188	311.203
Space group	Pnma (Orthorhombic)	P $\bar{1}$ (Triclinic)	P 2_1_/c (Monoclinic)
a (Å)	9.22 ± 0.01	9.696 (3)	9.061 (1)
b (Å)	10.76 ± 0.01	11.322 (4)	16.229 (3)
c (Å)	5.88 ± 0.01	7.076 (3)	11.322 (1)
α (°)	90	100.68 (4)	90
β (°)	90	93.40 (3)	108.68 (1)
γ (°)	90	107.04 (4)	90
*V* (Å^3^)	1575.41	724.468	1577.21
*Z*	4	2	4

### 2.2 Supramolecular arrangement

The supramolecular arrangements of the respective PQ salts were studied by normalized Hirshfeld surfaces (HS) (Spackman and Jayatilaka, 2009) and 2D fingerprint plots (Spackman and McKinnon, 2002) using the program CrystalExplorer17 (Turner et al., 2017). Then, the topological parameters were obtained by the quantum theory of atoms in molecules (QTAIM) (Bader, 1985; Bader, 1994) using the Multiwfn program (Lu and Chen, 2012). In QTAIM, the observable properties of the molecular system are contained in the electron density $\rho \left(r\right)$ of the molecular topology. The Laplacian of the electron density, $\nabla^{2}\rho$ , is a parameter that determines depletions and peaks of electron charge concentration between nuclear attractors in the molecular system topology, indicating the location of the bond critical points (BCP). In other words, $\nabla^{2}\rho$ indicates the concentration of electronic charge in the intranuclear region of two attractors: electronic density accumulated in the intranuclear region will result in a BCP with $\nabla^{2}\rho <0$ ; electronic density accumulated in the attractors (depletion in the BCP) will result in a BCP with $\nabla^{2}\rho >0$ (Bader, 1985; Matta and Bader, 2003). In the first case, the interaction is *shared*, such that the attractors are covalently bonded, while in the second case, the interaction is of the *closed-shell* type, in which the attractors are connected by weak electrostatic interactions (Bader, 1985; Bader, 1994). The topological parameters obtained by QTAIM are shown in Supplementary Table S1 (Supplementary Material S1). The results obtained low values of the electron density ( ρ< 0.1 au) and positive values of the Laplacian $\nabla^{2}\rho >0,$ indicating that the charge is depleted at the bond critical point (BCP). By the virial theorem,

$$\frac{1}{4}\nabla^{2}\rho \left(r\right)=2G\left(r\right)+\nu \left(r\right),$$ (1)

in atomic units, and by the expression,

$$h\left(r\right)=G\left(r\right)+\nu \left(r\right),$$ (2)

it was shown that the energy topological parameters are related to $\nabla^{2}\rho$ , where $h\left(r\right)$ corresponds to the electron density energy, $G\left(r\right)$ to the kinetic energy density, and $\nu \left(r\right)$ to the potential energy density. For H bonds, it was shown that the intensity of the interaction is very strong for $\nabla^{2}\rho <0$ and h<0 values, strong for $\nabla^{2}\rho >0$ and h<0 values, and weak or moderate for $\nabla^{2}\rho >0$ and h >0 values (Carroll and Bader, 1988). The binding energies ( BE ) (Emamian et al., 2019) were calculated using the formula,

$$BE\approx -332.34\rho \left(r\right)-1.0661,$$ (3)

where BE is given in kcal/mol.

## 3 Results and discussion

### 3.1 Molecular modeling analysis

**FIGURE 1:**
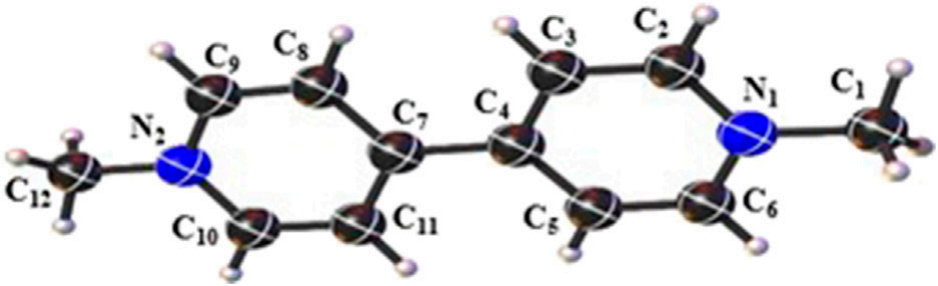
Solid-state *Ortep* representation of Paraquat, where the ellipsoids are drawn at the 50% probability level.

PQ consists of a quaternary BP structure (Figure 1) formed by the connection of two pyridine rings. In this structure, the N atoms are diametrically apart and, bonded in the *para*-position, a methyl group is present on each aromatic ring. The compound is produced in the form of a dichloride salt, where the organic part has two positive charges distributed along its chain.

In each of the crystals, the PQ salts were crystallized into distinct crystalline systems and space groups. The anhydrous salt was crystallized in the orthorhombic system, for which the space group is P*nma*; the structure of the dihydrate salt is found in the triclinic system and space group P $\bar{1}$ ; and, finally, the trihydrate form of the salt is found in the monoclinic system and space group P2_1_/*c*. PQC-I unit cell volume corresponds to 1575.41 Å^3^ and 19.9% of this total was calculated as void space (Supplementary Figure S1: Supplementary Material S1). In PQC-II and PQC-III, the unit cell volumes are filled by the chemical entities of the respective salts, in which in the latter, the excess H_2_O molecule raises the volume of the former in the proportion of 2.2:1. PQC-I, PQC-II, and PQC-III crystallographic data are shown in Table 1. In Section 3.2, other characteristics inherent to the crystalline structures of these salts will be discussed.

The PQC-II and PQC-III geometric parameters were compared with the PQC-I by the mean absolute deviation percent formula,

$$\text{MADP}=\frac{100}{n}\sum_{i=1}^{n}\left|\frac{\chi_{PQC-X}-\chi_{PQC-I}}{\chi_{PQC-I}}\right|,$$ (4)

where $\chi_{PQC-X}$ is the geometric parameters of the PQC-II and PQC-III and $\chi_{PQC-I}$ is the PQC-I geometric parameters. The graphs in Supplementary Figure S2 (Supplementary Material S1) show the comparison results carried out for the bond length and angle. The presence of water molecules does not significantly alter the BP cation structure. However, in PQC-III, we observed that the bond lengths are more sensitive to H_2_O, where the MADP value was 1.801%; in PQC-II, the MADP was 1.606%. H_2_O molecules were responsible for stretching the C_1_–N_1_ and C_12_–N_2_ bonds (on average 4.8%) while compressing the C_2_–C_3_ and C_10_–C_11_ bonds (on average 2.8%). On the other hand, the angles in PQC-II showed the greatest deviations, so the MADP value obtained was 0.885%, while in PQC-III, the MADP was 0.850%. Among others, the greatest variations occurred in C_5_–C_6_–N_1_ and C_8_–C_9_–N_2_ angles, whose average increase was 2.2%, except in the case of PQC-II, where the increase in the second was only 1.4%.

**FIGURE 2:**
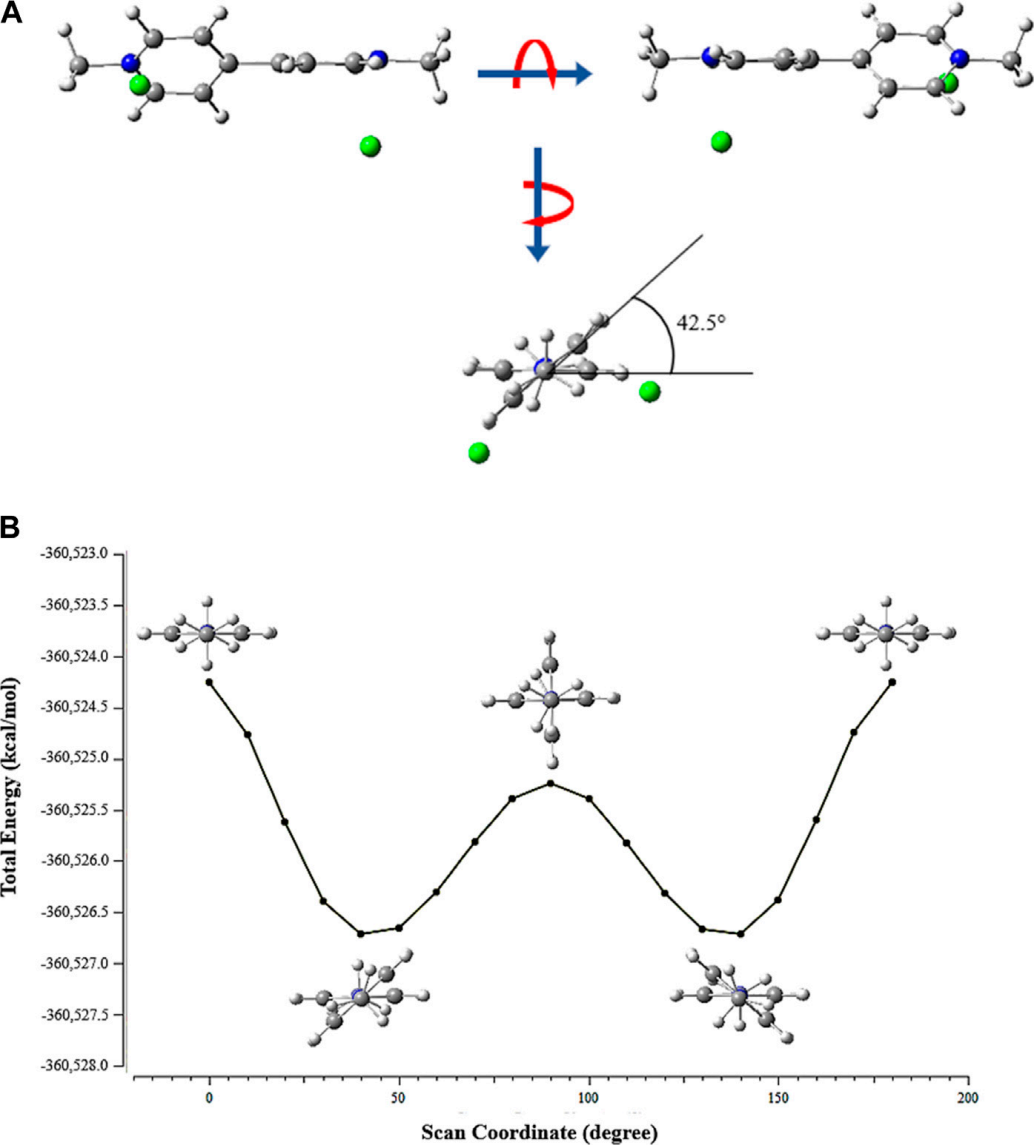
**(A)** The torsional effect on the bipyridylium cation structure by the presence of the Cl^−^ anions and **(B)** the relaxed scan of the energy due to the rotation of the C_4_−C_7_ bond by 180°.

The BP cation assumes a planar conformation in the crystals. However, in the gas phase and in the presence of Cl^−^anions, the calculations showed that its structure undergoes a torsion in the bond that joins the pyridylium portions, so that the planes formed by the aromatic rings meet at 42.5° (Figure 2A). The total energy scan showed that in conformations where the C_3_-C_4_-C_7_-C_8_ dihedral angle in the BP cation is 0° or 180°, the system is in the highest energy state (Figure 2B). However, the total energy is lower by rotating the aromatic portions by 40° and 140°.

**FIGURE 3:**
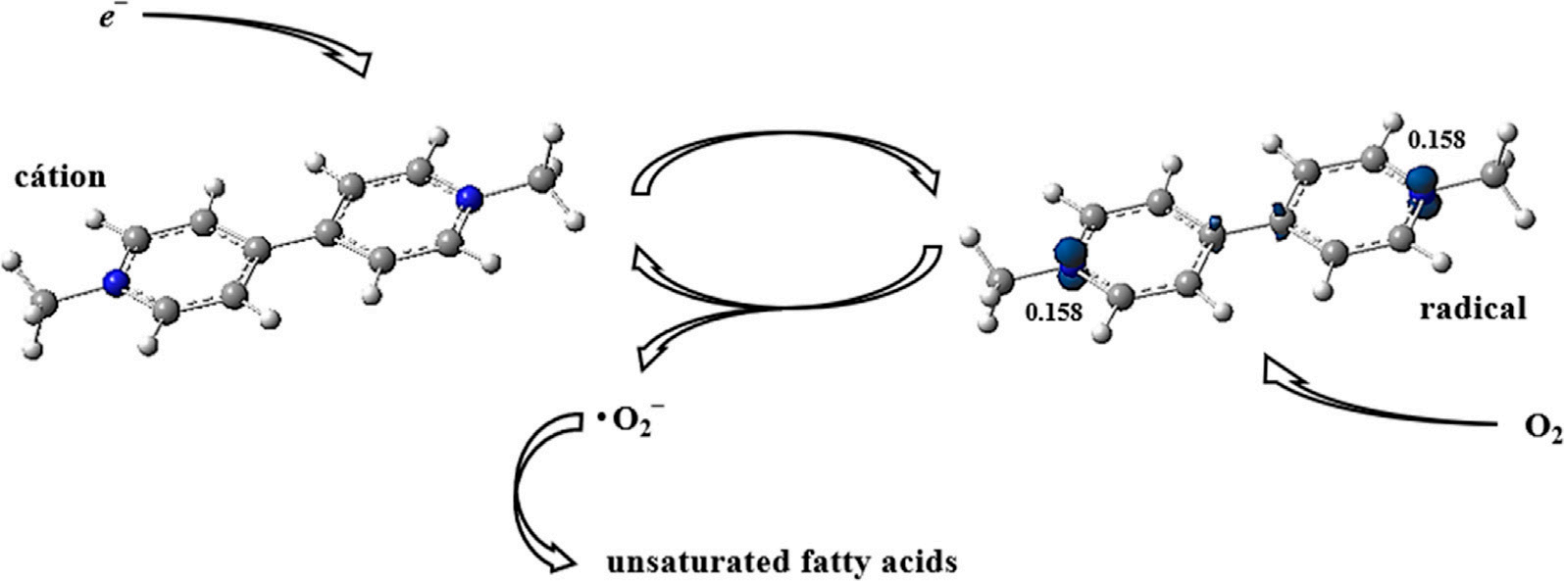
Paraquat’s mechanism of action on plant chloroplasts. The bipyridylium cation captures the electron produced in photosynthesis and becomes the free radical. The molecular oxygen present in the environment recovers the radical into a cation and transforms it into a superoxide radical, which destroys the unsaturated fatty acids, killing the plant.

FMO for the salt and BP cation are shown in Supplementary Figure S3. The respective HOMO and LUMO energies, as well as the energy gap (ΔE_H-L_), are shown in Table 2. According to Pearson’s principle, the FMO energy values indicated that the BP cation presents an acid character due to its low E_LUMO_ value. On the other hand, because of the presence of Cl^−^ions, salt has a markedly more basic character, which is justified by its high E_HOMO_ value. Furthermore, these data indicate that the BP ion is more susceptible to reduction during the weeds’ photosynthesis process. The high ΔE_H-L_ value for the cation, together with its high oxidation state, indicates a harder structure and, consequently, less polarizability. Chemical hardness

$$\eta =\frac{1}{2}{\left(\frac{\partial^{2}E}{\partial N^{2}}\right)}_{\upsilon \left(r\right)}=\frac{I-A}{2},$$ (5)

is an electronic property that measures the resistance to electron cloud deformation under small perturbations during chemical processes. In Eq. 5, E is the energy of the system, N is the number of particles, $\upsilon \left(r\right)$ is the external potential at point r , $I≅-E_{HOMO}$ is the ionization potential, and $A≅-E_{LUMO}$ is the electron affinity. The presence of the chloride anion in the salt reduces the BP cation’s ΔE_H-L_ value, allowing an electron cloud distortion in the presence of a momentary dipole; that is, the cation becomes more polarizable. In addition, the salt’s higher chemical potential allows charge transfer to lower chemical potential systems. Chemical potential

$$\mu ={\left(\frac{\partial E}{\partial N}\right)}_{\upsilon \left(r\right)}=-\frac{I+A}{2}=-\chi ,$$ (6)

is a measure of the charge transfer from a system of greater μ to one of smaller μ , and χ is the electronegativity. These values agree with the PQ redox processes in the chloroplasts, where the plant photosynthetic systems are contained (Photosystem I). In this environment, the electrons produced during the absorption of light energy are captured by the BP cation, resulting in the formation of a free radical. The results of the spin density calculations showed that the unpaired electron in the free radical is in the *p* orbitals of the N atoms (Figure 3), whose occupation is 0.84*e*, and the probability in each one is 0.158.

**TABLE 2:** Reactivity indices for bipyridylium cation, salt and radical, obtained at M06-2X/6-311++G(d,p) level of theory.

Descriptor	Cation (kcal/mol)	Salt (kcal/mol)	Radical (kcal/mol)
E_HOMO_	−387.60	−167.31	−286.14
E_LUMO_/E_SOMO_ ^a^	−222.32	−50.51	−198.41*
ΔE_H-L_ ^b^	165.28	116.81	87.73
Ionization Energy (*I*)	387.60	167.31	286.14
Electronic Affinity (*A*)	222.32	50.51	198.41
Electronegativity ( χ )	304.96	108.91	242.27
Chemical potential ( μ )	−304.96	−108.91	−242.27
Chemical hardness ( η )	165.28	116.81	87.73
Electrophilicity index ( ω )	281.33	50.77	334.52

^a^ In radical, E_SOMO_ (SOMO, singly occupied molecular orbital).

^b^ ΔEH-L = ELUMO–EHOMO.

During the process of plant photosynthesis, PQ is reduced to form a stable radical cation. Spin density calculations showed that the unpaired electron could be located equally on both nitrogen atoms of its structure. This cation rapidly reacts with the molecular oxygen present in chloroplasts, forming the superoxide ion from water molecules. From then on, other reactive oxygen species are formed, initiating lipid peroxidation, and culminating in the rupture of cell membranes.

### 3.2 Supramolecular arrangement description

**FIGURE 4:**
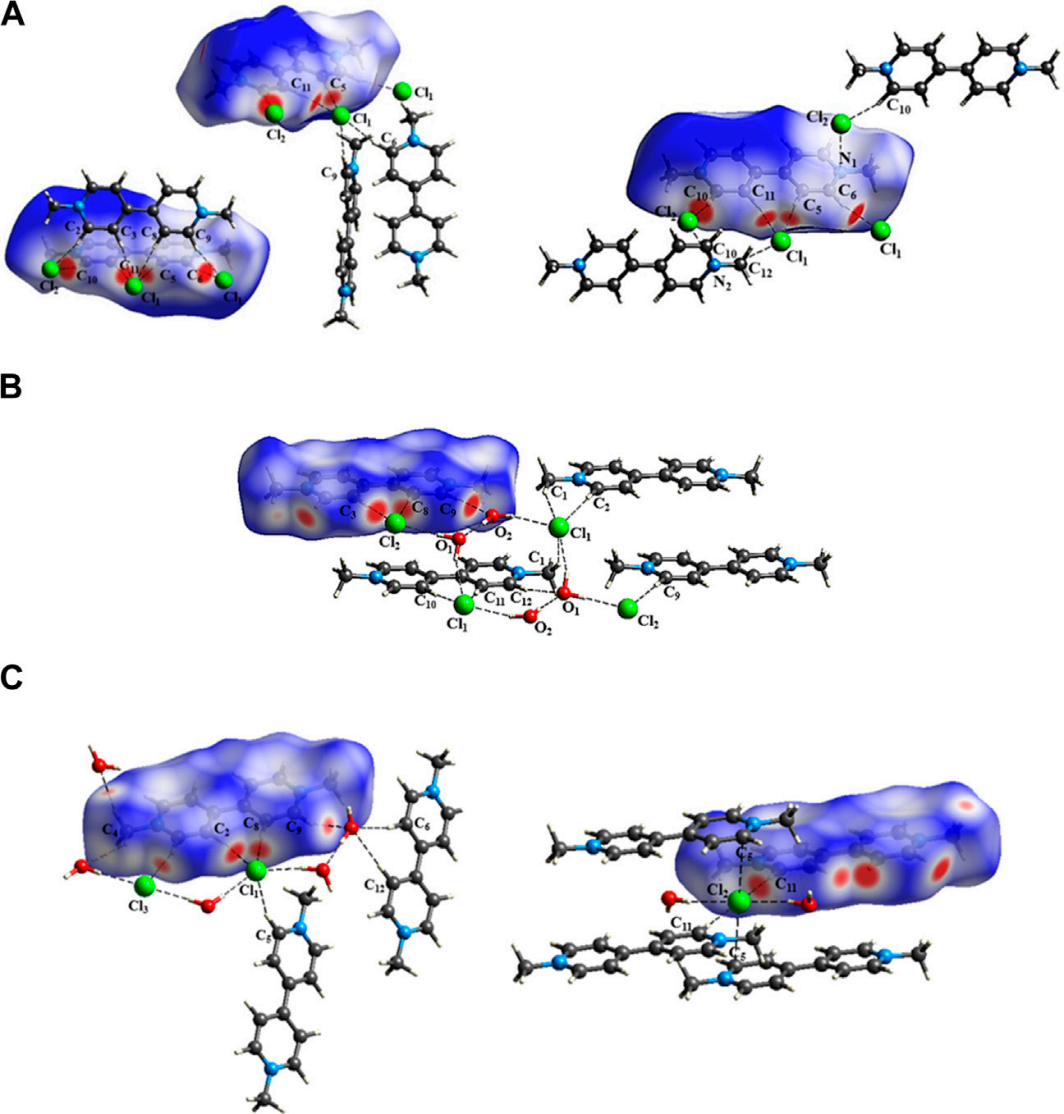
Hirshfeld surface $d_{norm}$ showing the intermolecular interactions in **(A)** PQC-I, **(B)** PQC-II, and **(C)** PQC-III supramolecular arrangements of the Paraquat salts. The red spots represent the short contact areas.

HS shows that, in the three crystal structures of PQ, the BP cation interacts with the Cl^−^ions as well as the water molecules in the hydrated salts at the same sites, as shown by the red circular regions (Figure 4). In these regions, the van der Waals spheres are superimposed, indicating short contacts, forming classical and non-classical H-bonds. The 2D fingerprint plots showed that the H⋯Cl contacts of the BP cation with the Cl^−^anions correspond to 19.2% of the HS in PQC-I, 15.3% in PQC-II and 11.0% in PQC-III. On the other hand, in PQC-III, the H⋯O interactions account for 9.8% of the HS, whereas in PQC-II, this area is just 5.8%. The topological parameters provided by QTAIM showed that, in all H⋯Cl and H⋯O interactions, the charge densities are very low (*ρ* < 0.1 a.u.) in the respective internuclear regions and $\nabla^{2}\rho >0$ , indicating that the electrons are depleted in the BCP and configuring *closed-shell* interactions. In these interactions, the nuclear attractors are connected by weak electrostatic interactions. Supplementary Table S1 presents the topological parameters obtained by calculating the structures of the PQ salts. It is notable that the number of interactions increases with the amount of co-crystallized water molecules in the salts.

**FIGURE 5:**
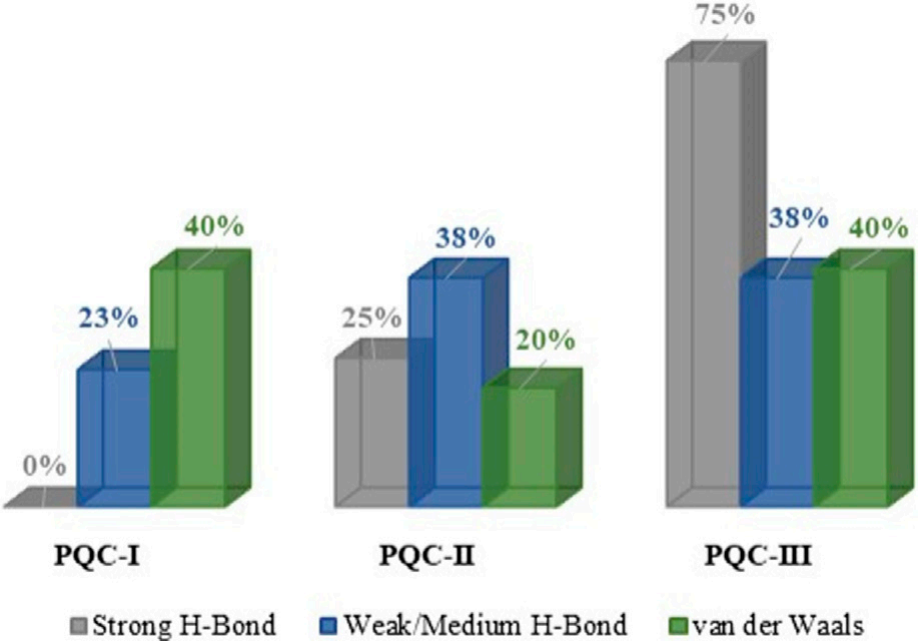
Percentage of each type of interaction occurring between the chemical entities in the Paraquat salts.

Furthermore, the total interactions accounted for in three salts (Figure 5) indicated that PQC-III held 75% of the strong H-bond (Hibbert and Emsley, 1990), being attributed to interactions O_3_–H⋯O_1_, O_3_–H⋯O_2_ and C_1_–H⋯O_2_, whose BE values are, respectively, −13.60, −17.62, and −13.36 kcal/mol. In PQC-II, the H atom bonded to C_1_ does not interact with O atoms. van der Waals interactions occur to a lesser extent in the PQC-II supramolecular arrangement, which is attributed only to C_5_–H⋯Cl_1_, where BE is −4.39 kcal/mol. However, the C_5_–H⋯Cl_1_ interaction also occurs in PQC-I, with BE being −6.45 kcal/mol, where, together with the topological parameters, it presents a weak/medium H-bond character. The C_2_–H⋯Cl_*x*_ interaction was observed in three crystalline environments. However, the associated energy increases in the order PQC-III < PQC-II < PQC-I, where the data point to a van der Waals interaction character in the trihydrate salt.

It was observed that in the interactions C_8_–H⋯Cl_*x*_ the BE values are similar in PQC-I and PQC-III (−7.51 and −7.05 kcal/mol). In PQC-I, the charge density in BCP is about 1.08 times greater, and, in addition, the slightly greater angle C_8_–H–Cl_*x*_ confers a more effective superimposition of the orbitals involved. In PQC-II, although this angle is quite favorable for orbital overlap, the Cl^−^ion is stressed in the structure, forming a structure like a pyramid with a “square” base, with the Cl^−^anion slightly below the plane of this base. However, the topological parameters indicated that in the three cases, C_8_–H–Cl_*x*_ is weak/medium H-bond. The C_10_–H–Cl_*x*_ interaction is very weak in PQC-I, showing a van der Waals character.

Finally, while the C_9_–H⋯O_*y*_ interaction in PQC-II is a strong H-bond, in PQC-III it is a van der Waals interaction, with the highest BE value in its supramolecular arrangement. This effect is because in PQC-III, the water molecule responsible for this interaction is strongly connected to two other water molecules, which in turn are connected to two Cl^−^ions, minimizing the contribution of the lone pair from O_3_.

### 3.3 Environmental impact

PQ is usually applied in the post-emergence of weeds in small concentrations (Cui et al., 2019), being absorbed by the foliage and binding strongly to organic and mineral matter, making it biologically inert. For this reason, PQ quickly became a hit on the market, given that the agriculturist could spray the weeds 1 day and sow the crop the next. PQ is poorly translocated within plants due to the rapid desiccation of plant tissues, so tubers and roots are not affected and can grow back. In addition, this herbicide is quickly absorbed by the soil, where the process of sorption is essentially ion exchange, and is deactivated, allowing new crops to be cultivated immediately without risk of phytotoxicity (Weber et al., 1965; Weber and Weed, 1968; Wibawa et al., 2009).

PQ also occurs in non-photosynthetic tissues, such as those of mammals. In these organisms, the compound is reduced through electron transfer in microsomes and mitochondria (Cochemé and Murphy, 2008), the mechanism being like that of photosynthetic systems. PQ is poorly absorbed through intact skin but can penetrate through skin wounds, which is of concern as the compound is a skin irritant (Tabak et al., 1990). Oral exposure is not considered relevant due to its low volatility; however, studies show that inhalation exposure may depend on climatic conditions. Oral exposure can occur through splashing in the mouth during mixing and transport, eating with contaminated hands, blowing on or sucking on spray nozzles, or eating contaminated food.

PQ’s toxic effects are observed not only in wildlife [terrestrial insects, birds, mammals, fish, algae, aquatic macrophytes, crustacean larvae, frogs (Eisler, 1990)], domesticated animal [cats, dogs, pigs, sheep, poultry, and geese (van Oers et al., 2005)], and human (Tsai, 2013) populations, but also in ecosystems. These include small lakes (Way et al., 1971) and reservoirs (Brooker and Edwards, 1973) from temperate and tropical regions, the latter including the Cerrado biome. PQ can thus harm non-target organisms (Martins, 2013), thus reducing biodiversity and the ecosystem services related to food security and farming profitability (Dennis et al., 2018). Furthermore, the intoxication of individuals can result in death, depending on ingested PQ concentration and species’ sensitivity; among vertebrates, mammals, including humans, are the most sensitive, displaying acute intoxication symptoms at 22–35 mg kg^−1^ body weight (Eisler, 1990; Huang et al., 2019). Intoxication can occur through bioaccumulation, expressed by injuries in the lungs (Tsai, 2013) and kidneys (McGwin and Griffin, 2022), and can contribute to Parkinson’s disease in humans (Zhang et al., 2016). However, PQ’s toxicity is not experienced only by vertebrates; it interferes with the habitat selection processes of fish (*Oreochromis niloticus* in this case), meaning that suitable habitats for fish with PQ concentrations higher than1.0 mg/L are avoided because of their low habitat quality, leading to the population’s decline (Soriwei et al., 2021).

Regarding the Cerrado biome, the contact of PQ with environmental biotic and abiotic components is facilitated by agricultural activity, resulting in low habitat quality and habitat loss when natural areas are converted to agricultural production (Schiesari and Grillitsch, 2011). However, the influence of PQ on the terrestrial environment is limited because of soil adsorption capacity associated with good agricultural practices. It is these practices and conditions that minimize the risk of causing pollution while protecting natural resources and allowing economically viable agriculture to continue, and in these conditions the use of PQ is not detrimental to soil-dwelling flora and fauna in the long term (Roberts et al., 2002). A similar situation is observed in the aquatic environment, where PQ’s availability is restricted because it is adsorbed by particles and sediment (Thi Hue et al., 2018). This situation seems to explain the few studies conducted to assess its toxicity for the environment in the Cerrado biome [influence of PQ on mortality of tadpoles (Lajmanovich et al., 1998) and native bees (Peruzzolo et al., 2021) and potential danger for freshwater species (Lundberg, 2021)], although the Brazilian Cerrado has been intensively used for agricultural purposes since the 1980s, involving the use of herbicides such as PQ. However, the current use of good agricultural practices in the Cerrado, such as no-till agriculture, seems not to prevent the environmental impacts of herbicides like PQ, because it aims for the expansion and profitability of large-scale farming based on input-intensive practices instead of sustainable agriculture processes (Ofstehage and Nehring, 2021).

## 4 Conclusion

The structure and reactivity of the BP cation, isolated and in PQ salts, were investigated, and theoretical data were used to understand the cation’s tendency for electronic capture during photosynthetic processes in chloroplasts, resulting in the formation of a stable free radical. The supramolecular arrangement structures of PQ salts showed that co-crystallization of H_2_O molecules leads to an increase in the number of strong interactions in the respective crystals. The Cerrado biome in central Brazil is composed of unique vegetation types that are a large source of bioactive compounds and provide great opportunities for sustainable agricultural practices. This biome has been used for agricultural purposes for some time, involving the use of the herbicide PQ until 2020, and the few studies conducted in Cerrado areas confirm its toxicity for the environment. While no decision has been made on the future use of PQ in Brazil, environmental studies based on legislation and physicochemical properties are essential to analyzing it within agriculture’s dynamic sector.

## Data Availability

The original contributions presented in the study are included in the article/Supplementary Materials, further inquiries can be directed to the corresponding authors.
