# Supplementary material for: The effect of water molecules on paraquat salts: from physicochemical properties to environmental impact in the Brazilian Cerrado
Source: Front Chem. 2023 Sep 19;11:1267634. doi: 10.3389/fchem.2023.1267634 (PMC10546189; doi:10.3389/fchem.2023.1267634)
Supplement: Supplementary file 1 [file DataSheet1.docx]

**The role of water molecules on Paraquat salts: from physicochemical properties to environmental impact in the Brazilian Cerrado**

**SUPPLEMENTARY Material**

| 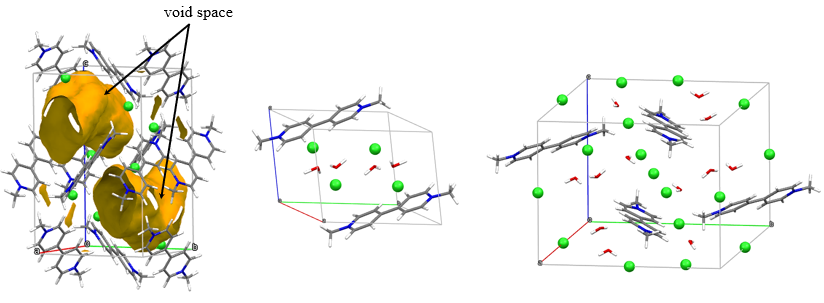 | | |
| --- | --- | --- |
| (a) | (b) | (c) |
| **Figure S1**. Void space in the unit cell of 1,1'-dimethyl-4,4'-bipyridylium dichloride. | | |

| 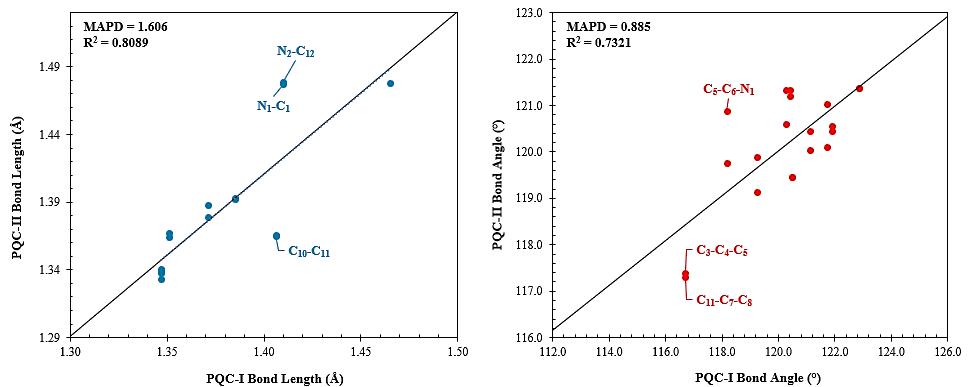 |
| --- |
| (a) |
| 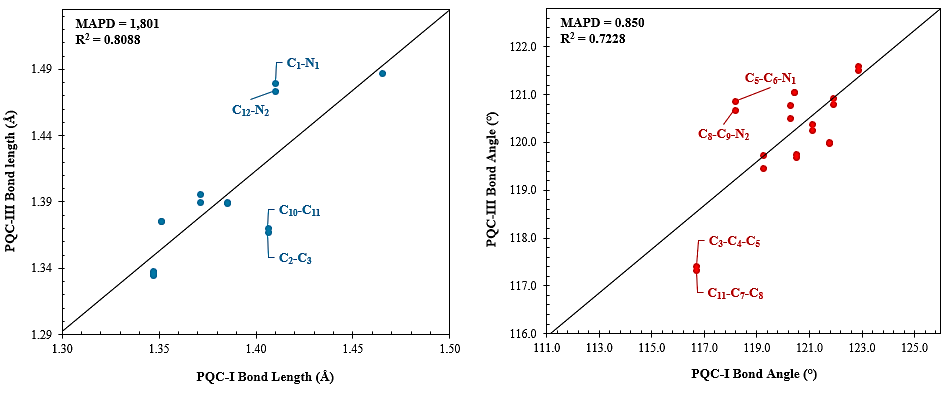 |
| (b) |
| **Figure S2**. Comparison graphs of the geometric parameters bond length and angle, obtained by XRD, for the (a) PQC-II and (b) PQC-III versus PQC-I. |

| 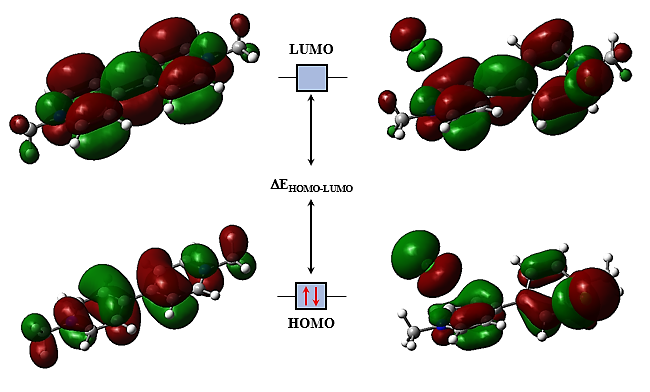 | | |
| --- | --- | --- |
| (a) |  | (b) |
| 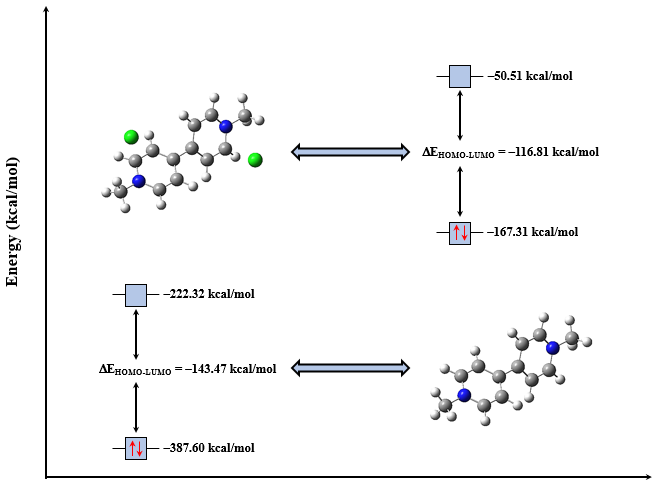 | | |
| (c) | | |
| **Figure S3**. HOMO and LUMO plots for (a) bipyridylium cation and (b) bipyridylium salt calculated at M06-2X/6-311++G(d,p) level of theory, and (c) the frontier molecular orbitals energy graph. | | |

| **Table S1**. Topological parameters at the bond critical point obtained by QTAIM for interactions in the supramolecular arrangement of PQC-I. | | | | | | | | | | |
| --- | --- | --- | --- | --- | --- | --- | --- | --- | --- | --- |
| **Interaction** | **H**$\boldsymbol{\cdots}$**A (Å)** | **D–H**$\boldsymbol{\cdots}$**A (°)** | **Topological parameters** | | | | | $\frac{\left\vert\boldsymbol{v}\left( \mathbf{r} \right) \right\vert}{\boldsymbol{G}\left( \mathbf{r} \right)}$ | **E_I_^(^*^f^*^)^ (kcal/mol)** | **Interaction Type** |
|  |  |  | $\boldsymbol{\rho}_{\boldsymbol{BCP}}\boldsymbol{(}\mathbf{r}\boldsymbol{)}$**^(^*^a^*^)^** | $\boldsymbol{\nabla}^{\mathbf{2}}\boldsymbol{\rho}_{\boldsymbol{BCP}}$**^(^*^b^*^)^** | $\boldsymbol{G(}\mathbf{r}\boldsymbol{)}$**^(^*^c^*^)^** | $\boldsymbol{v(}\mathbf{r}\boldsymbol{)}$**^(^*^d^*^)^** | $\boldsymbol{h(}\mathbf{r}\boldsymbol{)}$**^(^*^e^*^)^** |  |  |  |
| ***Anhydrous bipyridylium chloride*** | | | | | | | | | | |
| C_2_–H$\cdots$Cl_1_ | 2.738 | 166.26 | 0.0194 | 0.0603 | 0.0147 | -0.0142 | 0.0004 | 1.0 | -7.51 | weak/medium H-bond |
| C_3_–H$\cdots$Cl_1_ | 2.529 | 174.20 | 0.0147 | 0.0460 | 0.0105 | -0.0095 | 0.0010 | 0.9 | -5.95 | weak/medium H-bond |
| C_5_–H$\cdots$Cl_1_ | 2.461 | 169.89 | 0.0162 | 0.0529 | 0.0122 | -0.0113 | 0.0010 | 0.9 | -6.45 | weak/medium H-bond |
| C_8_–H$\cdots$Cl_1_ | 2.461 | 169.89 | 0.0194 | 0.0603 | 0.0147 | -0.0142 | 0.0004 | 1.0 | -7.51 | weak/medium H-bond |
| C_10_–H$\cdots$Cl_1_ | 2.378 | 166.29 | 0.0092 | 0.0288 | 0.0060 | -0.0048 | 0.0012 | 0.8 | -4.12 | van der Waals |
| C_11_–H$\cdots$Cl_1_ | 2.529 | 174.20 | 0.0063 | 0.0198 | 0.0039 | -0.0029 | 0.0010 | 0.7 | -3.16 | van der Waals |
| C_6_–H$\cdots$Cl_2_ | 2.348 | 162.75 | 0.0214 | 0.0613 | 0.0155 | -0.0157 | -0.0002 | 1.0 | -8.18 | weak/medium H-bond |
| C_9_–H$\cdots$Cl_2_ | 2.348 | 162.75 | 0.0214 | 0.0613 | 0.0155 | -0.0157 | -0.0002 | 1.0 | -8.18 | weak/medium H-bond |
| ***Bipyridylium chloride dihydrate*** | | | | | | | | | | |
| O_1_–H⋯Cl_1_ | 2.539 | 163.43 | 0.0215 | 0.0686 | 0.0157 | -0.0142 | 0.0015 | 0.9 | -8.21 | weak/medium H-bond |
| O_1_–H⋯Cl_2_ | 2.409 | 176.20 | 0.0248 | 0.0732 | 0.0177 | -0.0171 | 0.0006 | 1.0 | -9.31 | weak/medium H-bond |
| O_2_–H⋯Cl_1_ | 2.409 | 160.43 | 0.0213 | 0.0678 | 0.0154 | -0.0138 | 0.0016 | 0.9 | -8.14 | weak/medium H-bond |
| O_2_–H$\cdots$O_1_ | 1.954 | 165.21 | 0.0227 | 0.0575 | 0.0213 | -0.0282 | -0.0069 | 1.3 | -8.61 | weak/medium H-bond |
| C_10_–H$\cdots$Cl_2_ | 2.500 | 165.09 | 0.0159 | 0.0503 | 0.0106 | -0.0085 | 0.0020 | 0.8 | -6.35 | weak/medium H-bond |
| C_1_–H$\cdots$Cl_1_ | 2.774 | 157.38 | 0.0146 | 0.0424 | 0.0089 | -0.0072 | 0.0017 | 0.8 | -5.92 | weak/medium H-bond |
| C_2_–H$\cdots$Cl_1_ | 2.539 | 160.80 | 0.0166 | 0.0496 | 0.0105 | -0.0086 | 0.0019 | 0.8 | -6.58 | weak/medium H-bond |
| C_5_–H$\cdots$Cl_1_ | 2.633 | 172.68 | 0.0100 | 0.0273 | 0.0056 | -0.0044 | 0.0012 | 0.8 | -4.39 | van der Waals |
| C_11_–H$\cdots$Cl_1_ | 2.600 | 171.29 | 0.0163 | 0.0488 | 0.0102 | -0.0082 | 0.0020 | 0.8 | -6.48 | weak/medium H-bond |
| C_6_–H$\cdots$O_1_ | 2.380 | 169.24 | 0.0151 | 0.0528 | 0.0114 | -0.0097 | 0.0018 | 0.8 | -6.08 | weak/medium H-bond |
| C_8_–H$\cdots$Cl_2_ | 2.566 | 173.18 | 0.0139 | 0.0431 | 0.0090 | -0.0071 | 0.0018 | 0.8 | -5.69 | weak/medium H-bond |
| C_9_–H$\cdots$O_2_ | 2.255 | 165.07 | 0.0476 | 0.3425 | 0.0887 | -0.0917 | -0.0031 | 1.0 | -16.89 | strong H-bond |
| ***Bipyridylium chloride trihydrate*** | | | | | | | | | | |
| O_1_–H⋯Cl_1_ | 2.364 | 177.53 | 0.0272 | 0.1019 | 0.0274 | -0.0294 | -0.0019 | 0.9 | -10.11 | weak/medium H-bond |
| O_1_–H⋯Cl_3_ | 2.422 | 164.60 | 0.0222 | 0.0618 | 0.0145 | -0.0135 | 0.0010 | 1.1 | -8.44 | weak/medium H-bond |
| O_2_–H⋯Cl_1_ | 2.387 | 169.42 | 0.0270 | 0.1169 | 0.0284 | -0.0275 | 0.0009 | 1.0 | -10.04 | weak/medium H-bond |
| O_2_–H⋯Cl_2_ | 2.387 | 160.73 | 0.0257 | 0.0757 | 0.0200 | -0.0211 | -0.0011 | 0.9 | -9.61 | weak/medium H-bond |
| O_3_–H⋯O_1_ | 2.050 | 168.04 | 0.0377 | 0.3346 | 0.0889 | -0.0936 | -0.0050 | 1.0 | -13.60 | strong H-bond |
| O_3_–H⋯O_2_ | 1.999 | 175.11 | 0.0498 | 0.3282 | 0.0787 | -0.0754 | 0.0033 | 1.0 | -17.62 | strong H-bond |
| C_3_–H⋯Cl_1_ | 2.625 | 172.49 | 0.0164 | 0.0734 | 0.0146 | -0.0108 | 0.0038 | 1.3 | -6.52 | weak/medium H-bond |
| C_2_–H⋯Cl_3_ | 2.745 | 164.31 | 0.0122 | 0.0360 | 0.0073 | -0.0055 | 0.0018 | 1.3 | -5.12 | van der Waals |
| C_1_–H⋯O_2_ | 2.588 | 160.11 | 0.0370 | 0.0582 | 0.0183 | -0.0220 | -0.0037 | 0.8 | -13.36 | strong H-bond |
| C_6_–H⋯Cl_1_ | 2.601 | 160.12 | 0.0269 | 0.0420 | 0.0128 | -0.0152 | -0.0024 | 0.8 | -10.01 | weak/medium H-bond |
| C_5_–H⋯O_3_ | 2.355 | 170.75 | 0.0121 | 0.0421 | 0.0100 | -0.0094 | 0.0006 | 1.1 | -5.09 | weak/medium H-bond |
| C_8_–H⋯Cl_1_ | 2.671 | 169.21 | 0.0180 | 0.0860 | 0.0176 | -0.0137 | 0.0039 | 1.3 | -7.05 | weak/medium H-bond |
| C_9_–H⋯O_3_ | 2.542 | 156.92 | 0.0015 | 0.0064 | 0.0011 | -0.0007 | 0.0005 | 1.7 | -1.56 | van der Waals |
| C_10_–H⋯Cl_2_ | 2.607 | 165.75 | 0.0168 | 0.0539 | 0.0126 | -0.0118 | 0.0008 | 1.1 | -6.65 | weak/medium H-bond |
| C_11_–H⋯O_3_ | 2.521 | 177.44 | 0.0171 | 0.0591 | 0.0146 | -0.0145 | 0.0002 | 1.0 | -6.75 | weak/medium H-bond |
| ^(^*^a^*^)^Total electronic density on BCP; ^(^*^b^*^)^Laplacian of electron density on BCP; ^(^*^c^*^)^Lagrangian Kinect energy; ^(^*^d^*^)^Potential energy density; ^(^*^e^*^)^Total energy density; ^(^*^f^*^)^H-bond binding energy. | | | | | | | | | | |
